# A Cyclic Self-Calibration Method Based on a High-Precision Piston and Standard-Meter Water Flow Prover

**DOI:** 10.3390/s26092649

**Published:** 2026-04-24

**Authors:** Xuemei Geng, Yanwei Yang, Xuxin Dong, Xia Shou, Haiyan Ying, Qian Zhang, Cuiyi Zhang, Bin Zhou

**Affiliations:** 1 National Urban Energy Measurement Centre (Zhejiang Province), Zhejiang Institute of Quality Sciences, Hangzhou 310018, China; gengxuemei@sina.cn (X.G.);; 2 Key Laboratory of In-Situ Measurement of Ministry of Education, China Jiliang University, Hangzhou 310018, China

**Keywords:** flow prover, standard meter, cyclic self-calibration, in situ calibration, piston prover

## Abstract

The standard meter water flow prover is widely used due to its mature technology and high work efficiency. However, the process of sending the standard meter for calibration is not only time-consuming and laborious but also introduces deviations due to differences in installation and operating conditions. Therefore, by leveraging the high precision, convenient adjustment, and simple traceability of the piston prover, as well as the good repeatability of the standard meter, this paper proposed a cyclic self-calibration method, which iteratively transmits the calibrated piston value to different flow points of the standard meter through the standard meter itself and the transition flow meter, so as to realize the in situ transmission of the value from the high-precision piston to the wide range of standard meter. The water flow standard device and the cyclic self-calibration method, combining the standard meter and the transfer meter in series and parallel, were introduced; the uncertainty transfer chain was analyzed based on the error transfer theory; and experimental validation was conducted. The results show that the proposed self-calibration method is feasible, that the (0.005~4) m^3^/h high-precision flow rate of the piston can be extended to the (4~1250) m^3^/h of standard meters through multiple self-calibration value transfers, and that the expanded uncertainty of the water flow standard device is better than 0.20% (k = 2).

## 1. Introduction

The measurement performance of a water meter, an instrument for measuring tap water, is directly related to the vital interests of thousands of households [1]. According to a report by the China Research Institute of Industry, the demand for intelligent water meters in China in 2023 was approximately 55.8 million. Each water meter must be compulsorily calibrated in accordance with water meter calibration procedures. It will only be put into use on the market after calibration. In addition, the accuracy of the standard device should be better than 1/3 of the meter being tested. This means that a more accurate flow standard device is required when calibrating a high-precision flow meter [2]. However, many instrument manufacturers cannot afford the high cost of establishing high-precision and large-diameter flow standard devices, and it is difficult to meet the large number of rapid calibration needs of water meters [3].

Compared with the volumetric method and the mass method, the standard meter flow standard device is highly efficient and can expand the flow range by connecting standard meters in parallel, thus realizing the calibration of large-diameter flow meters [4]. However, the standard meter needs to be calibrated regularly, and the meter under calibration needs to be disassembled and sent to a professional calibration agency for calibration. The whole process not only has problems such as cumbersome disassembly and assembly, long distance for calibration, and long calibration time, but also introduces additional errors due to the mismatch between calibration conditions and usage conditions [5]. In contrast, in situ calibration is performed directly in the actual operating environment, which can avoid errors introduced by disassembling the machine for calibration and improve work efficiency. This efficient, convenient, and accurate calibration method has gradually become an inevitable trend in modern industrial flow meter calibration.

In situ calibration was initially implemented through a traceability chain, transferring measurement values from national standards with a small range to working standards with a large range [6,7,8]. Hernández Vásquez et al. proposed an indirect gravity measurement method, which uses a small volume and high-precision flow mass to infer the flow mass of a large volume of liquid to achieve high-precision dynamic flow measurement [9]. Oliver Büker et al. also used pistons as primary standards, equipped with four electromagnetic flow meters to cover different flow ranges, in combination with digital valves to generate dynamic flow, thus achieving traceable calibration of household flow meters under dynamic conditions, filling the technical gap between static calibration and practical application [10]. To enable automated batch calibration of water meters, Hankai Zhai et al. designed a highly automated and intelligent ultrasonic water meter flow calibration system, which is based on a method combining the standard meter and the weighing techniques [11].

On this basis, China has proposed the concept of equal-precision transfer. When a standard meter with good repeatability is used at a fixed point, its repeatability error will decrease as the number of measurements increases, and it may even approach zero [12]. However, in the actual value transfer process with finite repetitions, repeatability errors are inevitable and cannot be ignored. The random error introduced by repeatability is an important source of uncertainty, while the system deviation caused by the influence of the Reynolds number can be effectively compensated by the internal algorithm of the instrument. For the entire (0.005~1250) m^3^/h flow range, the corresponding Reynolds number range at 20 degrees Celsius is 1.18 × 10^4^~1.49 × 10^6^. The instrument will follow the preset characteristic curve, and its built-in real-time signal processing system can automatically adjust the instrument coefficient based on the real-time calculated Reynolds number, thereby realizing dynamic correction.

The transmission of flow value relies on the high-precision standard device as the primary standard to ensure the accuracy and reliability of measurement results [13]. The High-Pressure Piston Proofer (HPPP) has become one of the most advanced primary standard devices recognized internationally due to its short traceability chain, high accuracy, and simple structure [14]. Grinten et al. [15] of PTB used the HPPP as the primary standard device; in their study, it was passed step-by-step to the secondary standard and working standard composed of multiple turbine flow meters, thus solving the problem of calibration of large-diameter high-pressure natural gas flow meters. The Wuhan Branch of the National Petroleum and Natural Gas Large Flow Metering Station has established a high-pressure natural gas metering and calibration station consisting of HPPP primary standard devices, working standard devices, and mobile standard devices. The flow range is (20~9600) m^3^/h, and the expanded uncertainty is ≤0.25% (*k* = 2) [16].

However, the HPPP, as a passive piston, measures by pushing the piston through the fluid. Without a drive device, the flow cannot be adjusted quickly. The active piston device can rely on the motor to accurately control the movement of the piston to adjust the flow [17]. The traceability of active piston devices is established directly through length and time standards. While they can ensure the accuracy of high-precision flow meters, their applicable flow range is typically limited. Based on previous research, a water flow standard device that combines an active piston device with multiple standard meters is proposed in this paper. Through the cyclic self-calibration method, the value is transferred from the high-precision piston to a wide range of standard meters, thereby realizing in situ calibration of large-diameter flow meters.

## 2. Materials and Methods

### 2.1. Device Composition

**Figure 1:**
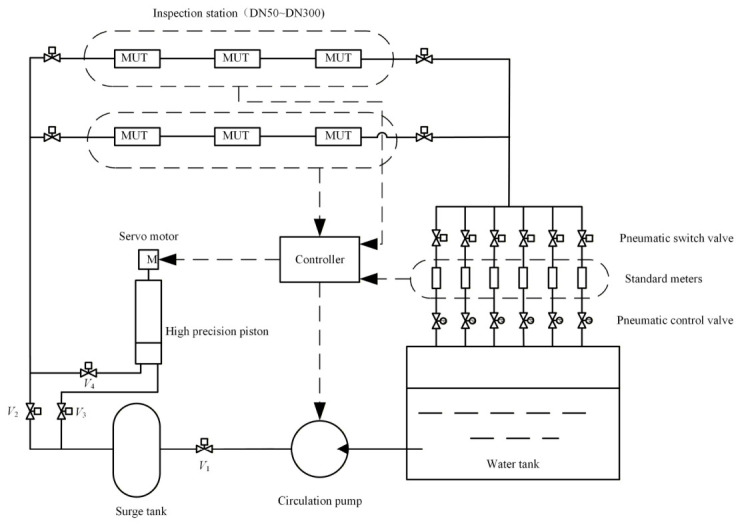
Schematic diagram of the water flow standard device system.

The water flow standard device mainly includes an active piston device, standard meters, a water tank, a water pump, experimental pipelines, and a control system (as shown in Figure 1). Based on the measurement principle, the piston device is both a flow source and a measurement standard. It is suitable for use as a traceability standard for a small range of flow meters, and its expanded uncertainty is 0.025% (*k* = 2). The standard meter system comprises five electromagnetic flow meters with an error within ±0.2% of full scale and one Coriolis mass flow meter with an error within ±0.1% of full scale (see Table 1). The maximum flow rate of the parallel standard meter can reach 1300 m^3^/h. During calibration, each station can connect two to six flow meters in series, providing a flexible configuration solution for the calibration of multiple flow meters.

The vertical cylindrical piston device primarily consists of a piston, a cylinder, and a driving mechanism. The nominal volume of the piston is 40 L, and the flow range is (0.005~4.0) m^3^/h. When the flow rate falls within the piston’s measuring range, *V*_1_, *V*_3_, and *V*_4_ are in operation, and the piston device is used to calibrate the meter under test. When working, the servo motor drives the screw assembly to rotate according to the set speed, driving the piston to move in a uniform straight line along the cylinder body, and records the displacement and movement time of the piston. The water discharged by the piston passes through the meter under test and is compared with the indicated value [18]. The relative measurement error *E* of the meter under test is:

(1) $$E=\frac{V_{m}/t_{m}-q_{p}}{q_{p}}\times 100\%$$

where *E* is the relative error, *V*_m_ is the cumulative volume of the meter under test, *t*_m_ is the cumulative test time of the meter under test, and *q*_p_ is the piston flow rate; the calculation formula is as follows:

(2) $$q_{p}=\frac{\pi }{4}D^{2}[1+2\alpha (T-293.15)]\times v$$

In the above formula, *D* is the effective diameter of the piston (20 °C), *α* is the linear expansion coefficient of the material, *T* is the water temperature in the piston cylinder, and *v* is the piston movement speed.

When the flow rate exceeds the piston range, valves *V*_3_ and *V*_4_ are closed, and *V*_2_ is opened. By adjusting the pump frequency and the control valve opening, the piston device is first used to calibrate the standard meter at the specified flow setpoints, after which the standard meter is employed to calibrate the MUT. When multiple standard meters are used in parallel, the flow measurement range can be further expanded. According to the continuity principle, by comparing the flow rate difference measured by the standard meter and the meter under testing in the same time interval, the error *E* of the meter under testing can be obtained as:

(3) $$E=\frac{q_{m}-q_{s}}{q_{s}}\times 100\%$$

where *q*_s_ is the flow rate of the meter being tested, and *q*_m_ is the flow rate of the standard meter.

The measurement error of the meter under testing is determined by comparing the volume flow rate flowing through it, while the Coriolis flow meter directly outputs the mass flow rate through the Coriolis effect generated, so the mass flow rate needs to be converted into volume flow rate. The density of water is the key parameter for converting mass to volume, and its accuracy directly affects the error and measurement uncertainty of the flow meter [19]. The density of water is affected by temperature, and its real-time density, measured by temperature, is as follows:

(4) $$\rho_{T}=\rho_{0}\times [1+\beta \left(T-T_{0}\right)]$$

where *ρ*_T_ is the liquid density at the current temperature, *ρ*_0_ is the liquid density at the standard temperature, and *β* is the expansion coefficient of water.

According to JJG643-2024 [20] “Verification Regulation of Flow Standard Facilities by Master Meter Method”, the combined uncertainty at a fixed flow point is affected by multiple factors, including the standard device, standard meter, timer, temperature, and pressure. When temperature or pressure changes occur, the volume of the fluid is altered, which in turn affects the flow measurement results. The impact of these factors on flow measurement can be evaluated through volume variations. Under standard conditions at 20 °C, a temperature change of 1 °C results in a relative volume change of approximately 0.02%, while a pressure fluctuation of 1 kPa leads to a relative volume change of about 4.6 × 10^−5^%. Simultaneously, timer errors are typically strictly controlled at the millisecond level, exerting a significantly lower influence on flow measurement compared to other factors. Overall, the uncertainties introduced by these factors are relatively small and can reasonably be neglected. Therefore, the combined uncertainty can be simplified to the following:

(5) $$u_{c}=\sqrt{u_{rs}^{2}+E_{R}^{2}}$$

where *u*_rs_ is the relative standard uncertainty of the standard meter at that flow point, and *E*_R_ is the repeatability of the meter being tested. Theoretically, as the result of independent measurement, the repeatability of each standard meter is also independent. However, in order to ensure the accuracy of the evaluation, according to the verification procedures, for the standard meter method with the flow standard device composed of multiple parallel standard meters, the maximum repeatability of the parallel standard meters is taken as the repeatability of the parallel standard meters. This is equivalent to assuming the error superposition under the most unfavorable conditions, avoiding the influence of correlation.

### 2.2. Self-Calibration Method

Because the flow range of the piston device is relatively limited and cannot meet the calibration requirements of large-diameter flow meters [21], it is necessary to combine the standard meter with the transfer meter and gradually expand the measured flow through the method of cyclic self-calibration until the set flow points are covered. During this process, the same flow meter may act as both a standard meter and a meter under testing at different stages.

**Figure 2:**
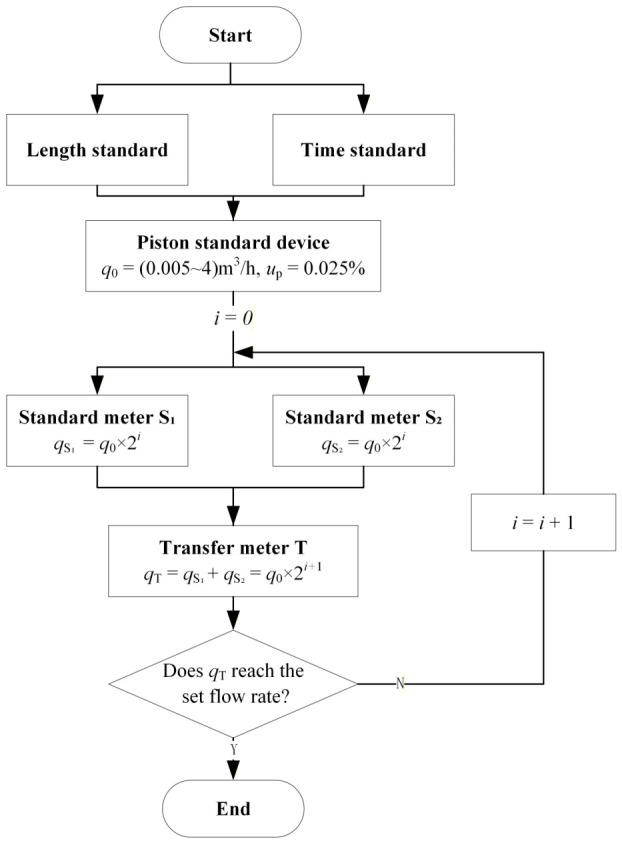
Schematic diagram of the cyclic self-calibration method.

As shown in Figure 2, taking the value transfer process between two standard meters S_1_ and S_2_ and the transfer meter T as an example, the entire value transfer process is mainly divided into four stages:

The first stage is flow value traceability. The piston standard device can accurately measure its displacement and time through the equipped grating ruler and timer. Its measurement results can be directly traced back to the length standard and time standard, with an uncertainty of up to 0.025%.

The second stage is the value transfer. We assume that the initial cycle number *i* is 0. By adjusting the regulating valve, the flow rate through the standard meter is *q*_0_ × 2^i^. At this time, the flow value is transferred to the *q*_0_ × 2^i^ flow point of the two standard meters by the piston device. That is to say, this flow point is calibrated by the piston standard device.

The third stage requires us to expand the flow. The flow value of the standard meter can be transferred to the transfer meter T through the standard meter method. However, due to the parallel connection of the standard meters, the flow of the transfer meter T is 2*q*_0_ × 2^i^. While the flow value is transferred through the traceability chain, the flow is also continuously expanding.

The fourth stage is cycle calibration. When the set flow point is not reached, it is necessary to expand the flow again under the premise of ensuring the flow value through the cyclic calibration method. That is, using the calibrated transfer meter T as the standard, the flow value is transferred back to the standard meters S_1_ and S_2_. At this time, the flow rates of the two standard meters are both 2*q*_0_ × 2^i^. Calibrations are repeated until the flow rate covers the set flow point.

It can be seen from Formula (5) that when the repeatability of the standard meter is smaller, the synthetic standard uncertainty is closer to the uncertainty of the upper-level standard device. The calibration of large-diameter flow meters requires continuous cycles to expand the flow range to the set flow point, with the value being transferred twice in each cycle. As the number of passes increases, the uncertainty also increases. According to the regulations, the maximum repeatability of the standard flow meter standard device is taken as the repeatability of the parallel standard meter. The simplified formula after *m* times of value transfer is

(6) $$u_{c}=\sqrt{u_{\mathrm{rs}}^{2}+mE_{R}^{2}}$$

The above formula shows that the combined uncertainty after multiple cycles of transfer is not only related to the repeatability of the standard meter, but also to the number of value transfers. Taking the starting flow point *q*_0_ = 4 m^3^/h as an example, the standard meter flow can be expanded to 1024 m^3^/h after 16 transfers. When the repeatability of the standard meter does not exceed 0.03%, the uncertainty of the maximum flow point is only 0.12%. When the repeatability of the standard meter does not exceed 0.05%, the uncertainty of the maximum flow point is 0.20%. This means that the greater the repeatability of the standard meter and the more times it is transferred, the greater the combined standard uncertainty. However, this uncertainty level is basically consistent with the accuracy grade of the standard meter, and it can meet the calibration requirements of most household and industrial water meters, see Table 2.

## 3. Results

Experimental tests were conducted for low-flow-rate points in the range of (4~45) m^3^/h. A DN15 Coriolis flow meter (CA15), a DN40 electromagnetic flow meter (AXG040), and a DN100 electromagnetic flow meter (AXG100) were selected as standard meters. The transfer standard meters were turbine flow meters manufactured by Shanghai Automation Instrumentation Co., Ltd., Shanghai, China, all with an error within ±0.2% of full scale. For the flow rate range of (4~20) m^3^/h, the LWGY40 model with a measurement range of (3~20) m^3^/h was adopted; for flow rates beyond this range, the LWGY80 model with a measurement range of (16~100) m^3^/h was employed.

**Figure 3:**
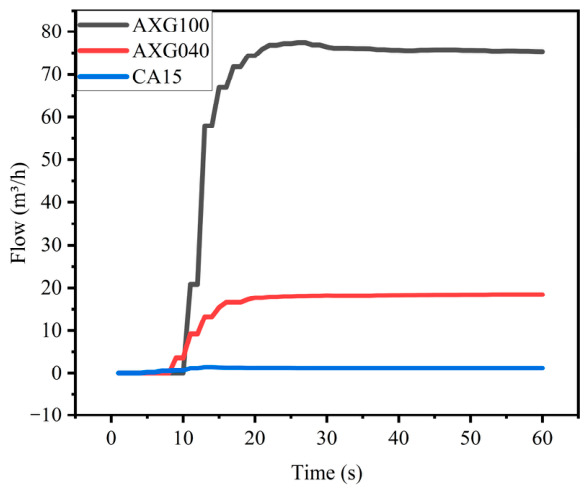
Flow rate trend curve.

In practical operation, due to the dynamic characteristics of the pipeline system, the flow rate typically exhibits certain fluctuations during the start-up and regulation processes. Under the operating conditions of a valve opening of 100% and a pump frequency of 15 Hz, continuous data were collected for 60 s from three standard meters, namely AXG100, AXG040, and CA15, see Figure 3.

The results show that the flow rates of the three standard meters undergo a pronounced increase with fluctuations in the initial stage, followed by gradual stabilization, reaching a steady state within 30 s. Although differences exist in the response amplitude and stabilization process among standard meters with different ranges, the overall trends are consistent.

Based on the above analysis, to ensure that data acquisition is conducted under stable conditions, a stabilization time of approximately 120 s is set during flow regulation, followed by a testing period of about 72 s after the flow has fully stabilized.

**Figure 4:**
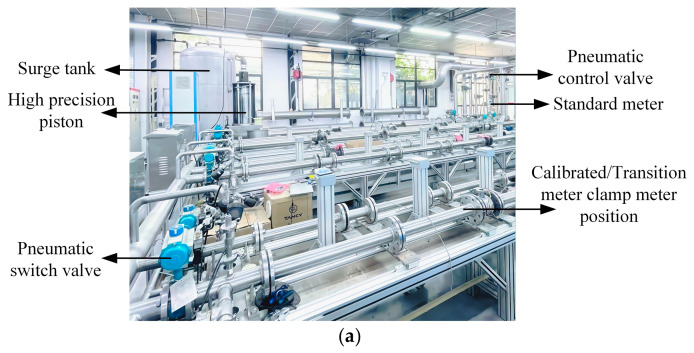

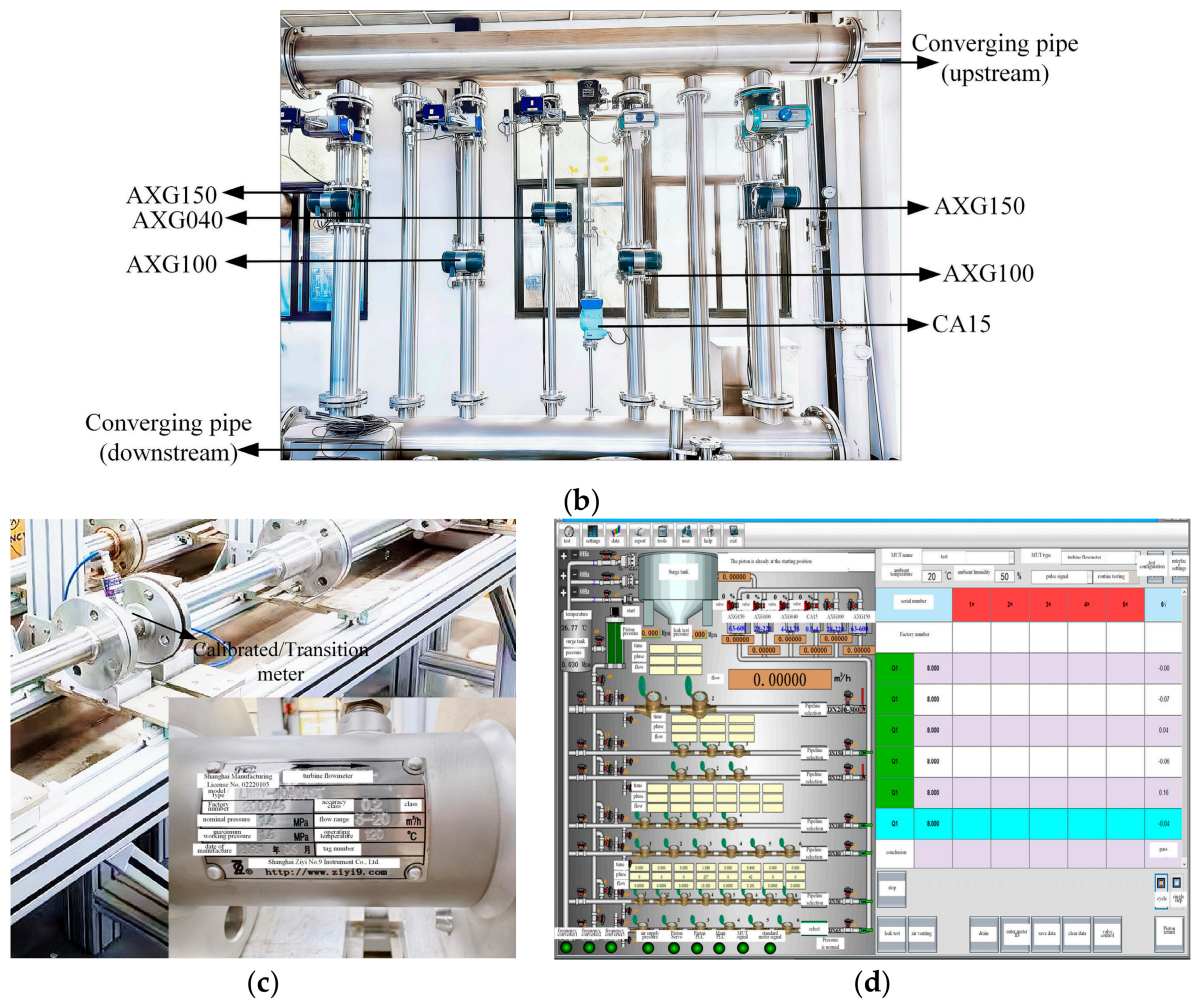
Water flow standard device experimental hardware and software: (**a**) Overall device; (**b**) standard meter; (**c**) calibration/transition meter; (**d**) software interface.

The flow regulation process is as follows: First of all, the pipeline should be leak-checked before calibration to avoid affecting the accuracy of device calibration. When calibrating small flow rate, one should open the valve corresponding to the piston device pipeline, set the flow point on the software, and wait for the device to automatically execute the process of returning the piston to the initial position and flow calibration. For flow points beyond the range of the piston, it is necessary to close the piston, start the water pump and its valve, set the frequency of the water pump inverter and the opening of the standard meter regulating valve on the software, and wait for the equipment to complete the water pump drainage and flow calibration process. Finally, the set flow point is reached through cyclic calibration between the standard meter and the meter being tested. Affected by the differences in pipelines and the coupling effect caused by the series and parallel connection of flow meters, there is a certain deviation in the calibration results. The adjustment standard must not exceed 5% of the set value, see Figure 4.

According to the above theoretical analysis, the value transfer is carried out with 4 m^3^/h as the starting flow point, and each transfer process is repeated six times. Considering that the upper limit flow of the mass flow meter is 4 m^3^/h, the mass flow meter CA15 is no longer reversely transferred in the subsequent cycle. As shown in Table 3, the flow value has reached the upper limit flow of LWGY40 when it increases from 4 m^3^/h to 20 m^3^/h, so it is necessary to replace the LWGY80 turbine flow meter as the transfer meter when transferring the 24 m^3^/h value. In order to finally realize the calibration of the electromagnetic flow meter AXG040 at the flow point of 45 m^3^/h, when the transfer meter transfers the flow direction to AXG040 and AXG100, it is necessary to adjust the opening of the regulating valve to stabilize the flow rate flowing through it at 23 m^3^/h (the adjustment deviation meets the adjustment standard of ±5%). At this time, the flow of the two parallel standard meters reaches 46 m^3^/h, and finally, the regulating valve is adjusted to realize the complete value transfer chain. After density compensation of the flow output by CA15 according to actual working conditions, it can be seen that, although there are slight differences in errors between different flow meters during the transmission process, the repeatability never exceeds 0.05%, which means it fully meets the standards for high-precision flow transmission and ensures the reliability of the experimental results.

The uncertainty of the four-time value transfer at different flow points is shown in Table 4. It can be seen from the data that the combined standard uncertainty of each flow point increases gradually as the flow rate increases from 4 m^3^/h to 45 m^3^/h. The combined standard uncertainty of the turbine flow meter at the flow point of 45 m^3^/h is only 0.129%, and the expanded uncertainty is 0.258% (k = 2). This result is close to the theoretical analysis results in Table 2, indicating that the high-precision cyclic self-calibration method is practical and feasible, and that it can effectively ensure high-precision transmission at different flow points.

## 4. Conclusions

In this study, a high-accuracy cyclic self-calibration method was proposed based on the traditional standard meter method. By introducing a piston prover as the primary standard and establishing a cyclic calibration framework between standard meters and transfer meters, the flow range can be progressively extended while maintaining measurement traceability and accuracy. Compared with conventional approaches, the proposed method enables in situ calibration of large-diameter flow meters without disassembly or off-site verification, thereby overcoming the limitations imposed by the range of a single standard device and flow meter. Through multi-stage transfer and parallel flow superposition, a significant expansion of the flow range was achieved.

Theoretical analysis indicates that, after 16 stages of value transfer, the flow range of the piston prover can be successfully extended from (0.005~4) m^3^/h to (4~1250) m^3^/h, with an expanded uncertainty better than 0.20%. Experimental results show that, after 13 transfer stages, the flow range is extended from 4 m^3^/h to 45 m^3^/h. The uncertainty increases with increasing flow rate, reaching a combined standard uncertainty of 0.129% at 45 m^3^/h, which agrees well with the theoretically predicted range of (0.12~0.14)%. These results verify the feasibility and accuracy of the proposed high-accuracy cyclic self-calibration method.

## Figures and Tables

**Table 1:** Summary of standard meter parameters.

Type	Brand Model	Quantity	Flow Range (m^3^/h)	Accuracy Level
Electromagnetic	Yokogawa AXG150 (Yokogawa Electric (Su-zhou) Co., Ltd., Suzhou, China)	2	63.62~636.2	0.2
Electromagnetic	Yokogawa AXG100 (Yokogawa Electric (Su-zhou) Co., Ltd., Suzhou, China)	2	28.27~282.74	0.2
Electromagnetic	Yokogawa AXG040 (Yokogawa Electric (Su-zhou) Co., Ltd., Suzhou, China)	1	4.52~45.24	0.2
Coriolis	Obar CA15-7011-SS (Hefei Oval Instrument Co., Ltd., Hefei, China)	1	0.5~4.0	0.1

**Table 2:** Cyclic self-calibration uncertainty at each flow point.

Number of Value Transfers	Flow Point (m^3^/h)	Uncertainty (%)	
		Repeatability Is 0.03%	Repeatability Is 0.05%
0	4	0.013	0.013
2	8	0.04	0.07
4	16	0.06	0.10
6	32	0.08	0.12
8	64	0.09	0.14
10	128	0.10	0.16
12	256	0.11	0.17
14	512	0.11	0.19
16	1024	0.12	0.20

**Table 3:** Self-calibration flow transfer experimental results.

Flow Point (m^3^/h)	Transfer Direction	Test Error (%)						Repeatability (%)
4	Piston > AXG040	−0.54	−0.54	−0.66	−0.55	−0.63	−0.62	0.05
4	Piston > CA15	−0.05	−0.04	−0.02	0	−0.01	−0.03	0.02
8	AXG040 + CA15 > LWGY40	0.04	−0.03	0.05	0	−0.07	0	0.05
8	LWGY40 > AXG040	0.02	−0.03	0.01	0.04	0.01	0.02	0.03
12	AXG040 + CA15 > LWGY40	−0.04	0.03	−0.02	0	0.01	−0.05	0.03
12	LWGY40 > AXG040	0.13	0.11	0.07	0.07	0.14	0.12	0.03
16	AXG040 + CA15 > LWGY40	−0.01	0.01	−0.07	−0.02	−0.1	−0.04	0.04
16	LWGY40 > AXG040	0.12	0.14	0.09	0.05	0.07	0.11	0.03
20	AXG040 + CA15 > LWGY40	−0.07	−0.03	−0.03	−0.08	−0.04	−0.06	0.02
20	LWGY40 > AXG040	0.1	0.04	0.1	0	0.04	0.01	0.04
24	AXG040 + CA15 > LWGY80	−0.01	0.01	0.03	−0.04	−0.04	0.02	0.03
23	LWGY80 > AXG040	0.06	0.1	0.04	0.12	0.07	0.02	0.04
23	LWGY80 > AXG100	−0.19	−0.14	−0.11	−0.16	−0.12	−0.15	0.03
46	AXG040 + AXG100 > LWGY80	0.16	0.19	0.2	0.21	0.2	0.2	0.02
45	LWGY80 > AXG040	−0.07	−0.03	−0.04	−0.1	−0.07	−0.04	0.03

**Table 4:** Self-calibration process value transfer relationship.

Number of Value Transfers	Flow Point (m^3^/h)	Transfer Direction	Combined Uncertainty (%)	Expanded Uncertainty (%)
1	4	Piston > AXG040	0.052	0.104
	4	Piston > CA15	0.024	0.048
2	8	AXG040 + CA15 > LWGY40	0.072	0.144
3	8	LWGY40 > AXG040	0.078	0.156
4	12	AXG040 + CA15 > LWGY40	0.084	0.168
5	12	LWGY40 > AXG040	0.089	0.178
6	16	AXG040 + CA15 > LWGY40	0.098	0.196
7	16	LWGY40 > AXG040	0.103	0.206
8	20	AXG040 + CA15> LWGY40	0.105	0.210
9	20	LWGY40 > AXG040	0.112	0.224
10	24	AXG040 + CA15 > LWGY80	0.116	0.232
11	23	LWGY80 > AXG040	0.123	0.246
	23	LWGY80 > AXG100	0.120	0.240
12	46	AXG040 + AXG100 > LWGY80	0.125	0.250
13	45	LWGY80 > AXG040	0.129	0.258

## Data Availability

Data are not publicly available and can be obtained by contacting the corresponding author if necessary.
